# Academy of Stomatology

**Published:** 1896-12

**Authors:** 

**Affiliations:** Academy of Stomatology


### ACADEMY OF STOMATOLOGY.

A MEETING of the Academy of Stomatology was held in Philadelphia, October 27, 1896, the President, Dr. James Truman, in the chair.

Upon the completion of routine business the President called for the report of the clinic held in the afternoon. This was read by Dr. Louis Jack, as follows:

### NOTES OF PROCEDURE IN TREATMENT OF DENTINAL SENSIBILITY BY ELECTRICAL OSMOSIS.

*Subject.*—R. L., second molar; cavity on buccal surface, at the cervix, extending below the gum.

*Condition.*—Caries, with acute sensibility.

*Apparatus.*—Chloride of silver dry-cell battery, with Willms's controller; fifteen cells.

*Obtundent.*—Cocaine hydrochloride, twenty-four-per-cent. aqueous solution.

There appeared to be difficulty in securing insulation of the tooth in the absence of a suitable clamp to detain the rubber dam below the cervical margin of the cavity. This was indicated by the ampèrage as recorded being much higher than the usual reading. Ordinarily the quantity of current through the dentine does not rise above three-tenths millii, rarely reaching four-tenths. The complete effect of the current sometimes is produced by an ampèrage of one-tenth millii.

In this case two applications were made. At the first the milammeter indicated one and three-tenths millii; during the second trial, one and two-tenths millii. This result was a clear indication of leakage of current through the contiguous tissues. Notwithstanding this the result of the first application reduced the sensibility to a tolerable degree, except at the distal border. After the second application of twelve minutes the relief was complete.

The case exhibits the importance of absolute isolation of the tooth by means of rubber dam, well secured, since two applications were required where one of probably a shorter duration would have been sufficient.

Notwithstanding the evident disadvantages labored under, the clinic was a satisfactory demonstration of the value of the method of effecting obtundation of acute dentinal sensibility by cocaine electrical osmosis.

After presenting the above report, Dr. Jack read the skeleton notes of forty cases in the order in which they have been treated since April 28 of this year.

(For notes, see page 783.)

The President then called upon the essayist of the evening, Dr. William H. Trueman, who read a paper on pyorrhœa alveolaris.

(For Dr. Trueman's paper, see page 774.)

DISCUSSION.

*The President.*—The subjects are now open for discussion.

*Dr. Burchard.*—This paper, as all others which Dr. Trueman has read to us, is interesting, particularly from an historical point of view. In regard to this matter of the history of this or any other branch of science, commentators frequently fall into error as to the end and purpose of the study of the special history. Do not understand me that I underrate the achievements and studies of the preceding generations of practitioners, but we may readily and easily overrate the force and basis of their opinions. There is nothing more common among those who spend their time in the study of the historical aspect of any science than the over-estimating the weight and importance of ancestral opinion. Those of us who have a penchant for the study of ancient literature might very readily call Democritus the father of the atomic theory instead of Dalton, or, again, might hold that some of the astronomers of the Alexandrian school were the authors of the heliocentric theory of the universe, or still again maintain that the science of bacteriology as applied to pathology is two hundred and fifty years old, when the world went germ mad; instead, we find that Copernicus, fifteen centuries after the Ptolemies, and Henle, two hundred and some years after Leuwenhoek, are given the honor of the theories named; and why? because they—Copernicus, Dalton, and Henle—did not base their opinions solely upon deduction, but furnished actual demonstration. It is demonstration, not the mere expression of opinion, which counts in science. If we note Dr. Trueman's exposition of the disease under discussion, as he states, founded upon opinions two hundred and one hundred years old, we are impressed at once with the great difference existing between the disease processes described and what to-day we call pyorrhœa alveolaris. Certainly at a time when mercurial poisoning and scurvy were common, these writers quoted included in their classifications these diseases; the therapeutics recommended clearly indicate this. To take up the discussion of the paper seriatim would require too much time; an hour and a half could easily be occupied in its direct discussion. He touches upon the etiology, clinical history, pathology, and therapeutics of the disease. As to the opinion that pyorrhœa has its incipiency in the variety of stomatitis described by Dr. Trueman, the necrotic stomatitis of children, his own proposition gives denial, for while stating that pyorrhœa is rare before middle life, he assigns as a probable cause a disease of childhood. The pathology of the disease under discussion is its interesting feature. We must start with the premise that pyorrhœa alveolaris is a generic term, naming but one symptom common to many diseases; but it has come to be applied to describe a tolerably constant set of morbid conditions, and if we are to give due credit for its description it should be given to Dr. Riggs, who first differentiated this disease from the many other conditions which did and do affect the teeth. At the present day, we may place the disease conditions which cause the loss of teeth through a molecular necrosis of pericementum under three heads: the first of these is where the disease begins at the gum margin and advances towards the apex of the root, causing progressive necrosis of the pericementum, and ultimately the loss of the tooth; the advance of the disease and also its early stages are marked by the deposition of dark scaly calculi, which follow the receding pericementum. The next class of cases are those causing the condition named by Dr. Darby gouty pericementitis; these begin upon some lateral aspect of the pericementum; with the margins of the gum unaffected, from a necrotic focus, the molecular death of pericementum radiates until the teeth are dislodged; commonly deposits are found in the necrotic focus, although they may not be; when pyogenic organisms gain entrance, pus formation is grafted upon the first necrosis. These are the cases in which a family and personal history of gout, rheumatism, rheumatoid arthritis, or lithæmia are elicited.

The next class are those described by Dr. Black, in the "American System of Dentistry," as phagedenic pericementitis, in my opinion one of the best and most descriptive titles in dental nomenclature. The pathology, clinical history, and prognosis bear a close relationship to the preceding class.

In the matter of therapeutics, Dr. Trueman has cited methods of treatment, applied in the past, which indicate that the disease conditions present were entirely different from any of those included under the three classes named.

In dealing with pyorrhœa we are not dealing with a specific disease, and therefore it is irrational and useless to look for a specific remedy. Specific remedies apply only to specific diseases, instead of having such a condition as implied in the latter. We are confronted with a tolerably constant set of surgical conditions, each of which calls for appropriate therapeusis. If we discover a cause, predisposing or exciting, this is, of course, removed; but besides this, there are conditions each of which requires appropriate treatment.

*The President.*—It is to be remembered that we have two subjects for discussion this evening,—the paper and records of Dr. Jack relative to cataphoresis, and this one of Dr. Trueman's. We have had much of pyorrhœa in the past year or two, and if no further discussion of that subject is desired, we will pass to the consideration of Dr. Jack's clinic and his records.

*Dr. Burchard.*—In view of the number of papers which have been read by Dr. William H. Trueman upon the history of dentistry, and his well-known interest and studies in that field, it no doubt impresses all of us that he is the one eminently qualified to give dentistry what it has not and what it needs,—a systematic history. If appropriate, I would suggest that the voice of this society express to him a conviction that it is actually his duty to do this work and to begin it at once.

*The President.*—That is very interesting and important, but it is not strictly in order at this time.

*Dr. Jack.*—There was one incident of great significance in the records which I have presented this evening, and that is, the effect of the cocaine in the case of pulp congestion cited in Cases XIII. and XVI. After the application of the current the approach to the pulp was made painlessly, the passage of the drill into the pulp-chamber denoted by the absence of resistance to the instrument was painless. The body of the pulp was removed without pain, but all attempts to extract the apical half of the pulp were futile, owing to the pain induced at every application of the broach. Further attempts to obtund by the cocaine solution and current were without avail, and an application of tannin and oil of cassia was made to the pulp remnant.

*Dr. Guilford.*—On an average, how much time do you allow for the inducement of the analgesic state?

*Dr. Jack.*—The records show an average of about twelve minutes. In some cases the time is slightly less; in others it is somewhat longer. It will readily be recognized that, although a great deal of time appears to be consumed in rendering the dentine anæsthetic, it is a relative and not an actual expenditure, for in many of the more obdurate cases, without cataphoresis, an hour might be expended in a fruitless attempt at proper excavation, and even then be unaccomplished. Under the cocaine cataphoresis the parts may be worked upon at pleasure within some fifteen or twenty minutes at the utmost.

*Dr. Guilford.*—In what percentage of cases do you resort to cataphoresis?

*Dr. Jack.*—Only in those cases which exhibit an acute or persistent resistance to the action of our common obtundents, the number of cases in which its application is imperative is limited to those which are acute and which from this contra-indicate the ordinary means of reducing sensibility. The great value of electrical osmosis consists in the fact that in all cases the effect extends deeply in the dentine, whereas in acute cases obtundents and escharotics are limited to comparatively superficial action.

*Question.*—Do you use guaiacol in combination with cocaine?

*Dr. Jack.*—No. Its odor is very objectionable, and the conductivity of the aqueous solution of the cocaine hydrochloride, and in some cases the citrate, permits the ready passage of the current. Guaiacol, as I understand its properties, is a more indifferent conductor than these solutions.

*Dr. Burchard.*—Dr. Jack, you spoke of the benefit of cocaine cataphoresis in connection with pulp-capping. I believe it is your practice to cap pulps which have been the seat of active hyperæmia.

*Dr. Jack.*—Yes; always, provided, of course, the vascular disturbance has not exceeded hyperæmia. I may say that increased attention as to dentinal sterilization is a feature of increasing importance in the conservative treatment of the pulp. I bestow greater attention and more time to sterilization than formerly.

*Dr. Roberts.*—I noted in the clinic to-day that, while there was a short circuit through the tooth, as shown by the high reading of the milammeter, there was still enough current passed through the tooth to produce complete anæsthesia. This seems to indicate that it is not necessary to completely insulate the tooth, or else the analgesia of the dentine is produced by the action of the cocaine introduced by way of the gums upon the nerves themselves before they enter the tooth. It is to be noted that the patient felt the negative electrode held in the hand.

*Dr. Register.*—Is there any advantage in using a saturated solution of cocaine, or are the best effects noted in any definite percentage?

*Dr. Jack.*—The twenty-four-per-cent. solution seems the most efficient, particularly in shortening the period of time required. Its efficiency may be due to its higher conductive power. Necessarily the conductivity of a strong solution of the salt would be greater than the conductivity of a weak solution of the salt.

*Dr. Register.*—I notice that you have used two salts of cocaine, the hydrochlorate and the citrate. Has either an advantage over the other?

*Dr. Jack.*—I have used both experimentally to determine their comparative efficiency, but I do not find any apparent difference in their effects upon dentinal sensitiveness.

*The President.*—I think Dr. Woodward must have some interesting data as to the value of the cataphoric current.

*Dr. J. A. Woodward.*—I have used electrical osmosis with cocaine solutions for obtunding sensitive dentine since May, 1896. A twenty-per-cent. aqueous solution of the hydrochlorate of cocaine is usually employed. Practically any strength of solution from saturation to five per cent. is effective.

The current is from a No. 11, thirty-five cell, constant current, galvanic battery, furnished by the Chloride of Silver, Dry-Cell Battery Company, of Baltimore, Md. The No. 11, twenty-five cell battery would be more convenient, as it is divided into groups of five cells. My practice has been to use seven or fourteen cells (equivalent to one volt per cell) to commence with. Should the current from fourteen cells, at the first contact point of the current-controller, cause too much sensation or pain, the number of cells is reduced to seven, and the current again turned on and slowly increased until the current from the seven cells can be passed through the tooth without any sensation. The key of the current-controller is then returned to the "start," and seven additional cells switched in the circuit, and the current again turned on. This is slowly increased until sensation to the current ceases, when the key of the controller is carried to the extreme of the contact points to drive the cocaine into the dentine for two or three minutes. By this method some cells can be successfully brought under control which will not bear the current from fourteen cells, the number generally used. All living teeth are sensitive to the electrical current in various degrees, some patients bearing twenty-five cells (twenty-five volts) without any discomfort, and others only seven. Seven cells have been sufficient, at the fourth contact point of the current-controller, to secure absence of sensitiveness in the dentine; more time than usual, of course, is required with so light a current. Pulpless teeth appear to bear any amount of current. I have frequently turned on them the whole thirty-five cells (thirty-five volts) of my battery without any sensation being experienced by the patient. Place a living tooth in that amount of current and the patient instantly flinches,—an excellent test for a living pulp. Up to the present time I have obtunded the dentine in seventy cases. I have not always succeeded in securing complete absence of sensitiveness in excavating, but have reduced it to a degree easily borne by the patient. For extremely sensitive teeth, for extremely sensitive patients, who either cannot or will not submit to the preparation of cavities in their teeth, electrical osmosis is at its best. In these cases the patient at the end of the sitting is as fresh and free from nervous fatigue as at its commencement, and as for yourself, the relief is so great that you will be astonished at the strain you have been subjected to in trying to secure satisfactory operations for this class of patients. The average time has been for fifty cases fifteen and a half minutes. When it is considered that these are the patients for whom an obtunder is positively demanded, the time of the whole operation is not very materially prolonged. Patients of this class are so glad to be relieved of pain that any reasonable addition of time is a matter of no consequence.

Adjourned.

HENRY H. BURCHARD,
*Editor Academy of Stomatology.*

