# Correction

**Published:** 1897-02

**Authors:** 


### CORRECTION.

In Dr. Woodward's remarks, on page 827 of December number, nineteenth line from top, for "cells" read "teeth."

